# Thermotropic and Barotropic Phase Behavior of Phosphatidylcholine Bilayers

**DOI:** 10.3390/ijms14022282

**Published:** 2013-01-24

**Authors:** Hitoshi Matsuki, Masaki Goto, Kaori Tada, Nobutake Tamai

**Affiliations:** 1 Department of Life System, Institute of Technology and Science, The University of Tokushima, 2-1 Minamijosanjima-cho, Tokushima 770-8506, Japan; E-Mails: goto@bio.tokushima-u.ac.jp (M.G.); tamai@bio.tokushima-u.ac.jp (N.T.); 2 Department of Material Science and Technology, Kochi National College of Technology, 200-1 Monobe-otsu, Nankoku, Kochi 783-8508, Japan; E-Mail: tada@ge.kochi-ct.ac.jp

**Keywords:** bilayer, lipid membrane, phase diagram, phase transition, phosphatidylcholine, pressure

## Abstract

Bilayers formed by phospholipids are frequently used as model biological membranes in various life science studies. A characteristic feature of phospholipid bilayers is to undergo a structural change called a phase transition in response to environmental changes of their surroundings. In this review, we focus our attention on phase transitions of some major phospholipids contained in biological membranes, phosphatidylcholines (PCs), depending on temperature and pressure. Bilayers of dipalmitoylphosphatidylcholine (DPPC), which is the most representative lipid in model membrane studies, will first be explained. Then, the bilayer phase behavior of various kinds of PCs with different molecular structures is revealed from the temperature–pressure phase diagrams, and the difference in phase stability among these PC bilayers is discussed in connection with the molecular structure of the PC molecules. Furthermore, the solvent effect on the phase behavior is also described briefly.

## 1. Introduction

Biological membranes consist mainly of phospholipid and protein molecules arranged in organized but flexible sheets. The phospholipid components form a closed bilayer aggregate, which provides a basic structure of biological membranes, because they are amphiphilic molecules. The most noteworthy property of phospholipid bilayers is to undergo the structural change of the bilayer, called a phase transition, in response to environmental changes of their surroundings such as temperature, pressure, salts, pH and solvents. The melting phenomenon of fatty-acid chains, that is, *trans-gauche* conformational change of phospholipid molecules in the bilayers with increasing temperature, is a representative phase transition. The transition has been analyzed a great deal by lipid researchers by means of various kinds of physico-chemical methods [1,2]: macroscopic approaches, such as differential scanning calorimetry (DSC); microscopic approaches, such as X-ray and neutron diffraction; and, approaches by statistical mechanics, such as molecular simulation [3].

Although a number of studies on the phase transitions of phospholipid bilayers have been reported [4], most of them are performed as a function of temperature and the concentration or additive concentrations under atmospheric pressure. There are only a few studies regarding pressure effect on the bilayers, and information on the phase behavior under high pressure is also lacking. Because phospholipid bilayers respond non-isotropically to changes in the environmental variables (temperature, pressure, *etc.*), it is interesting to examine the pressure effect on the bilayer. For example, in a gel state, the phospholipid molecule extends in the direction of bilayer normal (increase in membrane thickness) and shrinks in the direction of bilayer parallel (decrease in cross-sectional area), whereas the situation is reversed in the liquid crystalline state [5]. Therefore, the application of uniform and isotropic hydrostatic pressure on the bilayers brings about great mechanical fluctuation in them, and then the membranes change the phase state to take the most stable one under the pressure, which leads to a non-observable state under atmospheric pressure.

**Figure 1:**
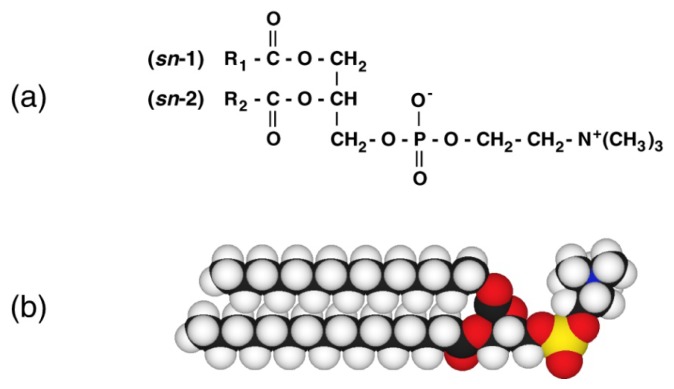
(**a**) Molecular structure of PC. (**b**) Three-dimensional picture of DPPC.

In many phospholipid species, the bilayer properties of phosphatidylcholines (PCs), which are major constituents in biological membranes of eukaryotic cells, have already been widely investigated [6,7]. The molecular structure of PC is shown in Figure 1a. On the other hand, studies on phospholipid bilayers under high pressure are considerably limited. Most of the pressure studies have concentrated on the bilayer properties of a certain phospholipid, dipalmitoylphosphatidylcholine (DPPC) [8–15], which is a symmetric PC with two equivalent palmitoyl chains as shown in Figure 1b. This seems to originate from the fact that DPPC has the maximum percentage in symmetric PCs (diacyl-PCs) in biological membranes such as erythrocyte membranes and that the bilayer is markedly desirable as model biological membranes because a gel-to-liquid-crystalline transition in the bilayer and an expanded–condensed transition in the insoluble monolayer can be easily observed at ambient temperatures experimentally. We have started the pressure study on phospholipid bilayers with the DPPC bilayer and investigated the phase behavior of various kinds of PC bilayers. In this review, we explain the bilayer properties of DPPC under atmospheric and high pressure in the first place, and then we show the thermotropic and barotropic phase behavior of bilayers of various kinds of PCs with different molecular structures. Furthermore, we also describe the solvent effect on the phase behavior of the DPPC bilayer.

## 2. Lyotropic, Thermotropic and Barotropic Phase Transitions of DPPC Bilayer

A solid crystal of a phospholipid has a well-ordered bilayer aggregate with lamellar structures [16]. When adding water to the solid crystal, water molecules hydrate the polar head group of the lipid molecule. The added water molecules are accumulated in the interlayer between bilayers and the lamellar structures are swollen. Since there is a limit of the thickness of the interlayer between bilayers (e.g., *ca*. 10 molecules for PC in the gel phase [17]), excess water molecules separate from the lamellar structures and become bulk water molecules. A bilayer aggregate formed by the hydration is a closed microsome with a diameter from about 20 nm to about 1000 nm, although the diameter is dependent on the molecular structure of the phospholipid and the preparation method. This closed bilayer aggregate with unilamellar or multilamellar structure formed by phospholipid molecules in aqueous solutions is referred to as a vesicle or a liposome.

**Figure 2:**
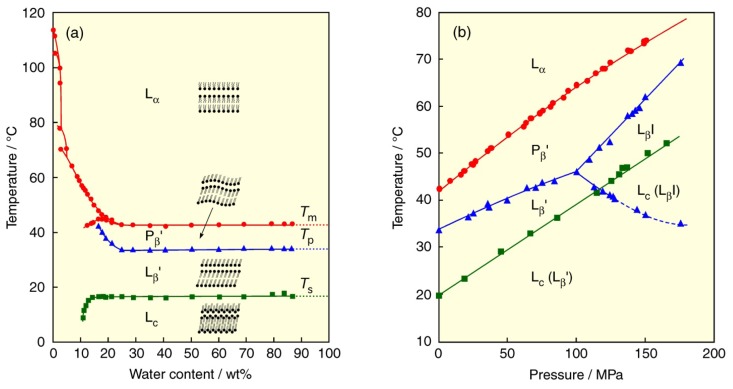
(**a**) Effect of water content on phase-transition temperatures of DPPC bilayer. The membrane state of each phase is also depicted in the figure. (**b**) Temperature–pressure phase diagrams of DPPC bilayer. Phase transitions: (green) subtransition, (blue) pretransition and transitions between gel phases, (red) main transition. Dashed line in figure (b) indicates the transition between metastable gel phases.

The bilayers of phospholipids undergo lyotropic phase transitions by hydration of water molecules. The lyotropic phase transitions of PC bilayers have been investigated in detail as a function of temperature [18,19]. The temperature–water content phase diagram of the DPPC bilayer is shown in Figure 2a together with the membrane state of each phase. The membrane states induced by hydration are: hydrated crystal or subgel (L_c_) phase, gel (L_β_) phase, and liquid crystalline (L_α_) phase. The melting point of nonhydrated crystal of DPPC (*ca.* 115 °C) drastically decreases with increasing water content, up to *ca.* 10% of water content. However, exceeding 10%, a phase transition originating from hydration change in polar head groups appears at a low temperature in addition to the chain melting transition at a high temperature. Furthermore, at 15% of water content, another phase appears in the gel phase at temperatures between the above two phase-transition temperatures due to the fluctuation of molecular packing in the bilayer. These phase transitions are called the sub-, pre- and main transitions from the low temperature and correspond to the transition between the L_c_ phase and the gel (lamellar gel (L_β_′)) phase, the transition between the gel (L_β_′ and ripple gel (P_β_′)) phases, the transition between the gel (P_β_′) phase and the L_α_ phase, respectively. Here, the superscript of prime means that lipid molecules are oriented on the tilt from the bilayer plane. The tilted orientation of lipid molecules in bilayers is one of characteristics for the bilayers of PCs with saturated acyl chains. Three kinds of phase-transition temperatures become almost constant, irrespective of water content beyond *ca.* 25%. Therefore, although lipid concentrations are considerably different in individual lipid-membrane studies, depending on the measurement methods such as DSC (>99% of water content) and X-ray diffraction (60%–95% of water content), the observed phase states in these methods are the same as each other.

In the region where the phase-transition temperatures are not affected by water content, we can specify the phase states of phospholipid bilayers. By adopting pressure (*p*) as an experimental variable together with temperature (*T*), we have investigated not only the thermotropic but also the barotropic phase transitions of various PC bilayers using DSC under atmospheric pressure and optical measurement methods under high pressure and constructed the *T*–*p* phase diagrams by using the obtained phase-transition temperatures and pressures. The detailed experimental techniques for the determination of the bilayer phase transitions and the discussion about thermodynamic quantities of the phase transition are described in the references given in the following sections. Figure 2b demonstrates the *T*–*p* phase diagram of the DPPC bilayer [20]. The three kinds of phase-transition temperatures increase linearly by applying pressure. A notable feature of the DPPC bilayer is the formation of nonbilayer structure, the pressure-induced interdigitated gel (L_β_I) phase, observed at high pressures above *ca.* 100 MPa. In the L_β_I phase, the acyl chains of the DPPC molecule on a monolayer of one side of a bilayer interpenetrate alternatively into those on a monolayer of the other side of a bilayer. Further pressurization brings about the extension of the L_β_I-phase region. The polymorphism of the gel phases such as the L_β_′ and P_β_′ phases with tilted molecular orientation and the nonbilayer L_β_I phase is characteristic of bilayers of PCs with a bulky choline head group. This is attributable to the large steric hindrance among adjacent choline groups.

## 3. Effect of Acyl Chain Structure on the Phase Behavior of PC Bilayer Membranes

So many species of phospholipids can be obtained by combining hydrophobic fatty-acid chains with different chain length or degree of unsaturation with several kinds of polar head groups. Here, we systematically explain the *T*–*p* phase diagrams of bilayers formed by various PCs that can be obtained by changing the molecular structure of DPPC.

### 3.1. Length of Acyl Chain

**Figure 3:**
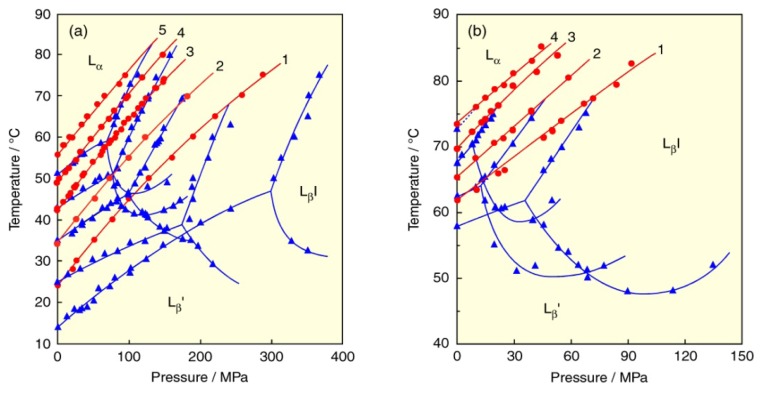
(**a**) Temperature–pressure phase diagrams of diacyl-PC (C*n*PC) bilayers: (1) C14PC (DMPC), (2) C15PC, (3) C16PC (DPPC), (4) C17PC, (5) C18PC (DSPC). (**b**) Temperature–pressure phase diagrams of C*n*PC bilayers: (1) C19PC, (2) C20PC, (3) C21PC, (4) C22PC. Phase transitions: (blue) pretransition and transitions between gel phases, (red) main transition.

The *T*–*p* phase diagrams for the bilayers of diacylphosphatidylcholines (C*n*PC: *n* = 14–18), which are symmetric PCs with saturated acyl chains of carbon number of *n* and homologs of DPPC (C16PC), are illustrated in Figure 3a [21]. Here the subtransition lines are omitted from the figure for simplicity. The phase diagrams of all C*n*PC bilayers are similar in shape although the diagram moves to the low-pressure and high-temperature region with increasing *n*. The similarity among the diagrams indicates that the slope (d*T*/d*p*) of each phase transition is similar to each other irrespective of the acyl-chain length. We revealed that the slopes of the phase boundaries depend on the kinds of the transitions and have similar values if the transition is the same [20–22]. With increasing acyl-chain length of the PC molecule, both the pre- and main-transition temperatures elevate [21–26]. Namely, the regions of the L_α_ and P_β_′ phase shrink (destabilize), while those of the L_β_′ and L_β_I phase extend (stabilize) in the order of the acyl-chain length of the PC molecule. It is understandable that the bilayer becomes more rigid in proportion to the magnitude of van der Walls interaction in the PC molecules. Furthermore, it is noted that the pressure of the triple point corresponding to the coexisting three gel (L_β_′, P_β_′, and L_β_I) phases steeply decreases with the chain elongation: the L_β_I phase remarkably extends the region to lower pressures. We call the pressure of the triple point the minimum interdigitation pressure (MIP).

We have recently examined the phase behavior of symmetric PCs with longer saturated acyl chains with a carbon number of 19 and over. Figure 3b shows the *T*–*p* phase diagrams constructed for the bilayers of symmetric PCs with saturated long acyl chains (C*n*PC: *n* = 19–22). With increasing acyl-chain length, the L_α_ and P_β_′ phases further shrink the regions while the L_β_′ and L_β_I phases further extend the regions and the MIP values decrease more and more. Interestingly, the phase boundary between the L_β_′ and L_β_I phases disappears in the case of the C22PC bilayer, and hence there is no triple point in the diagram. The phase behavior of the C22PC bilayer resembles those of ether-linked PC bilayers described in the following section. It can be said that the C22PC bilayer can induce the L_β_I phase even under atmospheric pressure. We confirmed the interdigitation of the C22PC bilayer under atmospheric pressure by the neutron scattering and fluorometry under high pressure [27]. The interdigitation of the C22PC bilayer by only hydration suggests that the attractive interaction between acyl chains due to the hydrophobicity is a dominant factor for the bilayer interdigitation, as well as the repulsive interaction among polar head groups.

**Figure 4:**
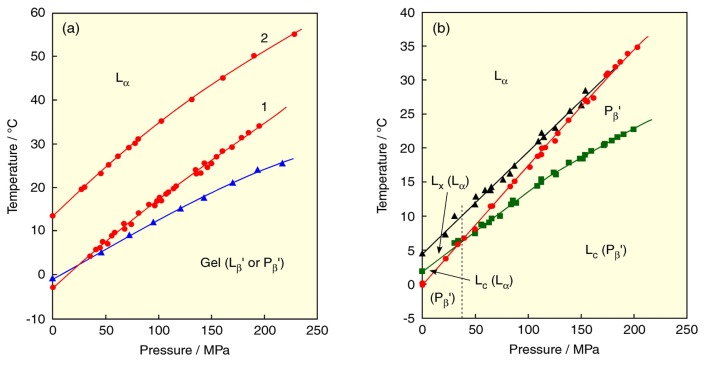
(**a**) Temperature–pressure phase diagrams of C*n*PC bilayers: (1) C12PC (DLPC), (2) C13PC. Phase transitions: (blue) pretransition of C13PC bilayer, (red) main transition. (**b**) Temperature–pressure phase diagrams of C12PC bilayer in 50 wt.% EG solution. Phase transitions: (green) subtransition, (blue) pretransition and transitions between gel phases, (red) P_β_′/L_α_ or P_β_′/L_x_ transition, (green) L_c_/L_x_ or L_c_/P_β_′ transition, (black) L_x_/L_α_ transition.

Meanwhile, we have also investigated the thermotropic and barotropic bilayer phase behavior of symmetric PCs with shorter saturated acyl chains. Our consideration on the thermodynamic quantities of the bilayer phase transitions revealed that the main transition occurs in the bilyers of PCs with acyl chains of a carbon number of 12 and over [22] and the bilayer interdigitation does in those of PCs with the chains of a carbon number of 14 and over [21]. Figure 4a depicts the *T*–*p* phase diagrams of symmetric PCs with shorter chains (C*n*PC: *n* = 12, 13) [21,22]. Unlike the bilayers of PCs with longer chains, the L_α_ phase region is more extended due to the depression in the main-transition temperature. The pretransition is no longer observed in the C12PC bilayer. Since it is reported [28,29] that the C12PC bilayer exhibits different phase behavior from other C*n*PC bilayers, we have investigated the behavior by using aqueous ethylene glycol (EG) solutions [30]. Here, EG was used as an antifreeze in order to avoid the water freezing, because the C12PC bilayer undergoes the main transition at a temperature below 0 °C. The phase diagram of the C12PC bilayer in 50 wt.% EG solutions is drawn in Figure 4b. An interesting feature of the C12PC bilayer is the existence of an intermediate liquid crystalline (L_x_) phase, which is thought to be a nontilted, partially disordered L_α_ phase, between the P_β_′ and L_α_ phases. The C12PC bilayer undergoes three kinds of transitions in EG solutions, in contrast with that in water, which undergoes only the main transition. Since the slope of the curve for the transition from the L_c_ phase is smaller than that of the main transition curve, both curves cross each other at 40 MPa on the diagram. We identified the former transition at pressures below 40 MPa as the L_c_/L_x_ transition and above 40 MPa as the L_c_/P_β_′ transition. The higher-temperature transition in EG solution is the transition from the L_x_ phase to the L_α_ phase. The transition coincides with the main transition at about 180 MPa. The stability of the L_x_ phase decreases gradually by pressure. At high pressures above 180 MPa, the P_β_′ phase transforms into the L_α_ phase directly.

### 3.2. Asymmetry of Saturated Acyl Chain

Most phospholipids contained in biological membranes of eukaryotic cells are not symmetric phospholipids with two equivalent acyl chains but asymmetric phospholipids with two different acyl chains [31]. We next describe the effect of the chain asymmetry on the phase behavior of PC bilayer membranes. Here we take up asymmetric saturated PCs with the chain difference of carbon number of 2 between the *sn*-1 and *sn*-2 chains, 1-palmitoyl-2-stearoyl-PC (PSPC) and its positional isomer 1-stearoyl-2-palmitoyl-PC (SPPC), 1-myristoyl-2-palmitoyl-PC (MPPC) and its positional isomer 1-palmitoyl-2-myristoyl-PC (PMPC). Two hydrophobic acyl chains in the *sn*-1 and *sn*-2 positions of symmetric PCs like DPPC are not originally equivalent with each other, although they have the chains of the same length. Since the *sn*-2 chain bends at the C2–C3 position in the molecule, the *sn*-2 chain becomes virtually 1.5 carbon–carbon bond lengths shorter at the end of the acyl chains than the *sn*-1 acyl chain if they take *all-trans* conformation in the gel phase [31,32]. Accordingly, the terminal methyl groups of both chains in the molecule are not aligned. In the case of PSPC (or MPPC) and SPPC (or PMPC), the distance at the terminal methyl ends between the *sn*-1 and *sn*-2 chains are 0.5 and 3.5 carbon–carbon lengths, respectively.

**Figure 5:**
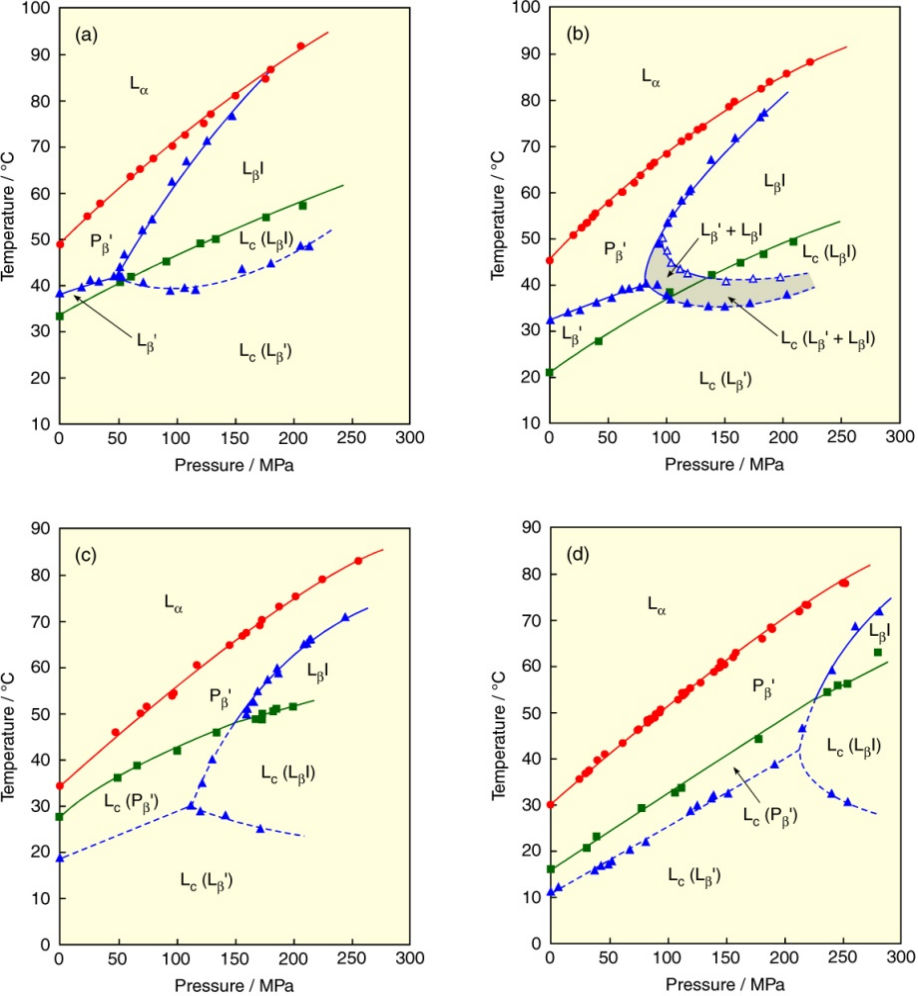
Temperature–pressure phase diagrams of bilayers of asymmetric PCs with saturated acyl chains: (**a**) PSPC, (**b**) SPPC, (**c**) MPPC, (**d**) PMPC. Phase transitions: (green) subtransition, (blue) pretransition and transitions between gel phases, (red) main transition. The gray-colored area in the figure of SPPC bilayer is the coexistence region between the L_β_′ and L_β_I phases. Dashed line in each figure indicates the transition between metastable gel phases.

Figure 5 depicts the *T*–*p* phase diagrams of the PSPC, SPPC, MPPC and PMPC bilayers [33–35]. The three phase transitions (sub-, pre- and main) can be observed in all bilayers in a manner similar to that of the DPPC bilayer, although the L_β_′ phase of the MPPC and PMPC bilayers turns to the metastable phase. All bilayers induce the L_β_I phase at a certain pressure irrespective of the chain asymmetry. The three transition temperatures of the PSPC or MPPC bilayer are larger than the corresponding bilayers of the positional isomer, SPPC or PMPC, respectively. This is because SPPC or PMPC with large chain asymmetry may reduce the stabilization of the L_c_ and gel phases in the bilayer because of the instability for a part of the chain difference between the *sn*-1 and *sn*-2 chains [36,37].

By contrast, in the PSPC or MPPC bilayer, the effect of the alignment of terminal methyl groups in acyl chains appears in the subtransition and the interdigitation: the alignment causes a significant elevation of the subtransition temperature and a marked depression of the MIP value. We can say that the membrane states with dense packing, like the L_c_ and L_β_I phases, definitely reflect the degree of the alignment of terminal methyl groups in acyl chains. In general, it is well known that the L_c_ phase formation of the bilayers of symmetric PCs with saturated acyl chains is very slow [26] and the formation requires a thermal treatment called annealing before experiments. On the other hand, the MPPC and PMPC can form the L_c_ phase easily on account of the metastability of the L_β_′ phase, in particular, the L_c_ phase formation of the MPPC bilayer is very fast and the fast formation enables us to study the state of the L_c_ phase in detail by a method of high-pressure fluorometry [38]. Moreover, there is a two-phase coexistence region (gray-colored area in the figure) of the L_β_′ and L_β_I phases in the SPPC bilayer. We elucidated from the high-pressure fluorometry [34,39] that the region results from a less polar “pocket” in the membrane, which is formed from the chain asymmetry in the terminal parts of acyl chains of SPPC.

### 3.3. Unsaturated Acyl Chain

**Figure 6:**
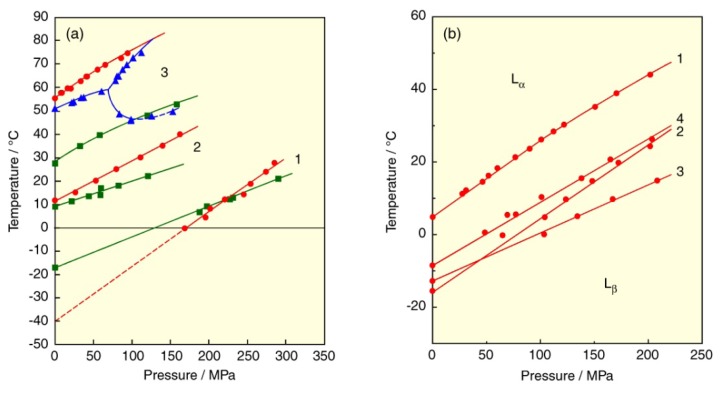
(**a**) Temperature–pressure phase diagrams of bilayers of symmetric PCs with unsaturated acyl chains: (1) DOPC, (2) DEPC, (3) DSPC (for comparison). (**b**) Temperature–pressure phase diagrams of bilayers of asymmetric PCs with an unsaturated acyl chains in the *sn*-2 position: (1) SOPC, (2) SLPC, (2)SAPC, (3) SDPC. Phase transitions: (green) subtransition or L_c_/L_α_ transition, (blue) pretransition and transitions between gel phases, (red) main transition. Dashed lines in figure (**a**) indicates the unstable main transition (curve 1) and the transition between metastable gel phases (curve 3).

Biological membranes contain a high percentage of phospholipids with unsaturated fatty acids. For example, about half of the phospholipids in human erythrocyte membranes have unsaturated fatty acids such as oleic, linoleic, arachidonic and docosahexaenoic acids, *etc.* [3]. We next explain the effect of chain unsaturation on the phase behavior of PC bilayers. We take up two symmetric unsaturated PCs, dioleoyl-PC (DOPC) and dielaidoyl-PC (DEPC), which have two fatty acids with a carbon number of 18 and a double bond (*cis* or *trans*) at the C9–C10 position in each chain. The *T*–*p* phase diagrams of the DOPC and DEPC bilayer membranes [40,41] are drawn in Figure 6a together with that of the bilayer of distearoyl-PC (DSPC: C18PC) that is a symmetric saturated homolog with the same carbon number. We can immediately notice that the bilayers of unsaturated PCs do not undergo the pretransition (transition between the gel phases) unlike the DSPC bilayer. This is because the acyl chains of unsaturated PCs become bulky in the presence of double bonds, which hinders the tilted orientation in the bilayer. Then, the DOPC and DEPC bilayers undergo phase transitions among the L_c_, L_β_ and L_α_ phases. We determined the transitions in reference to the results of experiments in 50 wt.% EG solutions [40–42] whereby the DOPC bilayer undergoes the L_c_/L_α_ at low pressures or sub- (L_c_/L_β_) transition at high pressures, and the main transition at all pressures, while the DEPC bilayer does the subtransition and the main transition at all pressures.

Comparing the main-transition temperatures of two kinds of unsaturated PC bilayer membranes under atmospheric pressure with that of the DSPC bilayer membrane, the temperatures are 55.6 °C for DSPC, 11.1 °C for DEPC and −40.3 °C for DOPC. The substitution of two *cis* unsaturated chains for two saturated chains of DSPC produces the great depression of the main-transition temperature by about 48 °C per chain. The introduction of two *trans* double bonds to both chains is equivalent to almost half of the effect of two *cis* bonds. In the gel state of the *cis* unsaturated PC bilayers, the packing of the bilayer was disordered by the effect of the unsaturated chain because a *cis* unsaturation induced a bend of acyl chain. This leads to the reduction of van der Waals interaction between PC molecules and the gel phase is destabilized [3,43]. The destabilization of the gel phase by *trans* unsaturation is weaker than that by *cis* unsaturation because acyl chains with a *trans* double bond are relatively straight in the gel phase [44–46]. To keep high fluidity in biological membranes, it is essential that the membrane state is the L_α_ phase. The great stabilization of the L_α_ phase by introducing unsaturated fatty acids with a *cis* double bond to a lipid molecule is well correlated with the fact that membranes of living things in extreme conditions, such as deep sea, contain a high percentage of unsaturated phospholipids. A similar trend is observed for the bilayer transitions related to the L_c_ phase under atmospheric pressure, that is, the subtransition temperatures are 28.2 °C for DSPC [26], 9.0 °C for DEPC and the L_c_/L_α_ transition temperature is −12.0 °C for DOPC. Unlike the main transition temperature, the temperature difference is not equivalent among all PC bilayers due to the difference in the kinds of the transition, this means that the structure of acyl chain also affects the stability of the L_c_ phase and the effect is stronger in a *cis* double bond than a *trans* bond.

In the DOPC bilayer, the main-transition curve intersects the curve of the transition related to the L_c_ phase at about 234 MPa, and the stability of gel (L_β_) phase changes at the intersection point: the L_β_ phase changes from metastable to stable at the pressure. This means that the L_β_ phase at a low pressure becomes rather unstable, and only the L_c_/L_α_ transition can be observed under atmospheric pressure [47]. Here great care should be taken in dealing with a phase transition of unsaturated phospholipids at a low temperature below the freezing point of water. The low-temperature phase transition of unsaturated phospholipid bilayers is generally regarded as the main (gel-liquid crystal) transition because only one transition is observed at a low temperature. However, at a temperature below the freezing point of water, the bilayers are prone to become the L_c_ phase because the bulk-water molecules freeze and simultaneously the molecules pull out the water molecules from the interlamellar region between the bilayers. And then, the L_β_ phase becomes metastable or unstable, and the observed phase transition is the L_c_/L_α_ transition, not the main transition as seen in the phase diagram of the DOPC bilayer. We have also examined the phase transitions of a series of *N*-methylated dioleoylphosphatidylethanolamine (DOPE) bilayers [42] and proved that the low-temperature phase transition of these DOPE bilayers is the L_c_/L_α_ transition, although the transitions have been generally considered to be the main transition.

### 3.4. Asymmetry of Unsaturated Mixed Acyl Chains

**Figure 7:**
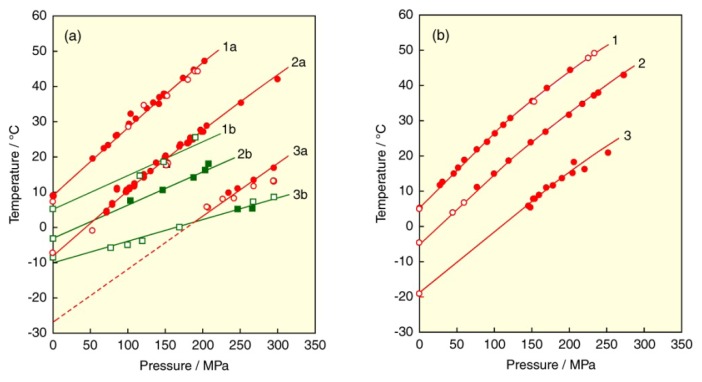
(**a**) Temperature–pressure phase diagrams for bilayers of PCs with an unsaturated acyl chain in the *sn*-1 position: (1a, 1b) OSPC, (2a, 2b) OPPC and (3a, 3b) OMPC. (**b**) Temperature–pressure phase diagrams for bilayers of PCs with an unsaturated acyl chain in the *sn*-2 position: (1) SOPC, (2) POPC, (3) MOPC. Open symbol refers to transitions in aqueous 50 wt. % EG solution. Phase transitions: (green) L_c_/L_β_ transition (1b) and L_c_/L_α_ or L_c_/L_β_ transition (2b, 3b), (red) main transition. Dashed line in figure (a) indicates the unstable main transition (curve 3a).

As mentioned in the previous section, most phospholipids of biological membranes have asymmetric acyl chains. Especially, they have an unsaturated chain in either *sn*-1 or *sn*-2 position. A few reports have been made on phase transitions of PCs with mixed acyl chains of a saturated and an unsaturated one [48–50]. We selected six kinds of mixed-chain PCs with an unsaturated acyl chain in the *sn*-1 or *sn*-2 position, 1-oleoyl-2-stearoyl-PC (OSPC), 1-oleoyl-2-palmitoyl-PC (OPPC), 1-oleoyl-2-myristoyl-PC (OMPC) and 1-stearoyl-2-oleoyl-PC (SOPC), 1-palmitoyl-2-oleoyl-PC (POPC), 1-myristoyl-2-oleoyl-PC (MOPC). The corresponding *T*–*p* phase diagrams of these mixed-chain PC bilayers are depicted in Figure 7 [40,41,51,52]. The bilayers of the mixed-chain PCs with an unsaturated acyl chain at the *sn*-1 position, OMPC, OPPC and OSPC, undergo two phase transitions, whereas those of the PCs with an unsaturated acyl chain at the *sn*-2 position, MOPC, POPC and SOPC, undergo one phase transition. In our previous studies [51,52], we identified from the thermal behavior and the pressure dependence of the phase-transition temperatures that the transitions observed in the OMPC and OPPC bilayers are the L_c_/L_α_ or sub- (L_c_/L_β_) transition and the main (L_β_/L_α_) transition and those observed in OSPC bilayer are the subtransition and the main transition, while the transition observed in MOPC, POPC and SOPC bilayers is the main transition. It is clear that the phase behavior of the bilayers of unsaturated mixed-chain PCs is greatly dependent on the binding position of an unsaturated acyl chain on the glycerol backbone.

The phase-transition temperatures of the bilayers of all unsaturated mixed-chain PCs increase with applying pressure and also with an increase in chain length of saturated acyl chain in the *sn*-1 or *sn*-2 position. For the OMPC, OPPC and OSPC bilayers, the pressure dependence of the main-transition curve is larger than that of the curve of the transition related to the L_c_ phase. With a decrease in the chain length of saturated chains in the *sn*-2 position, the main-transition curve intersects the curve of the transition related to the L_c_ phase, and the stability of the L_β_ phase changes at the intersection point. Accordingly, while the L_β_ phase exists as a stable one in the whole range of pressure in the OSPC bilayer, the L_β_ phase changes from metastable to stable at *ca.* 50 MPa in the OPPC bilayer and *ca.* 190 MPa in the OMPC bilayer. In the case of the OMPC bilayer, since the L_β_ phase at a low pressure becomes unstable as the case of the DOPC bilayer [47], it is difficult to observe the main transition under atmospheric pressure.

The main-transition temperatures of the OSPC and SOPC bilayers are 8.7 °C and 6.7 °C, respectively. This means that the effect of the introduction of a *cis* double bond to the *sn*-2 chain is slightly larger than the effect of the introduction of that to the *sn*-1 chain [52]. It is known that most of naturally occurring asymmetric phospholipids are phospholipids with a saturated chain at the *sn*-1 position and an unsaturated chain at the *sn*-2 position. We must therefore consider the effect of the polyunsaturation in the *sn*-2 acyl chain on the bilayer phase behavior. The *T*–*p* phase diagrams for the bilayers of SOPC, 1-stearoyl-2-linoleoyl-PC (SLPC), 1-stearoyl-2-arachidonoyl-PC (SAPC) and 1-stearoyl-2-docosahexaenoyl-PC (SDPC), which contain the stearate chain in the *sn*-1 position and the mono- or poly-unsaturated acyl chain in the *sn*-2 position, are shown in Figure 6b [53]. These unsaturated PC bilayers undergo the main transition under a normal condition. The transition temperatures of all bilayers increase linearly by applying pressure. The transition temperatures of the SOPC, SLPC, SAPC and SDPC bilayers under atmospheric pressure were 6.7, −15.0, −13.0 and −9.0 °C, respectively. The results suggest that the phase-transition temperature of unsaturated PC bilayers does not decrease monotonously with an increasing degree of unsaturation. Although the introduction of the first *cis* double bond into DSPC (yielding SOPC) lowers the phase transition temperature by 48.9 °C and two double bonds (SLPC) lowers it by a further 21.7 °C, four and six double bonds (SAPC and SDPC) bring about no further decrease, and unexpectedly cause a slight increase in the transition temperature. We have thermodynamically elucidated from the smallest d*T*/d*p* value of the SAPC bilayer that the SAPC bilayer has the gel phase with the loosest packing among these PC bilayers. On the other hand, the relatively high temperature of the main transition of the SDPC bilayer suggests that in the gel state, the bilayer exists in a rather regular and relatively stable conformation as compared to the other bilayers [54,55]. Since the acyl chain of SDPC must presumably be assumed to take a conformation approximately parallel to that of the bilayer normal, and motional freedom is severely limited by the lack of rotation at the six double bonds, allowed conformations are highly restricted. One of the possible conformations of SDPC in the gel state is as follows: the saturated *sn*-1 chain is well stretched, and the marked effect of the *cis* double bond in the *sn*-2 chain, including the reduction in the effective chain length, yields an approximately helical conformation because the hydrophobic chain of docosahexaenoic acid can take the conformation [56].

## 4. Effect of Linkage of Glycerol Backbone on the Phase Behavior of PC Bilayer Membranes

Diacyl-PCs are phospholipids, of which each of the two hydrophobic chains binds to the glycerol backbone by an ester linkage, and they are called ester-linked phospholipids. On the other hand, there exist other kinds of phospholipids, of which the hydrophobic chain binds to the glycerol backbone by an ether linkage, in the cell membranes of some organisms [57] and they are called ether-linked phospholipids. Plasmalogens (1-alkenyl-2-acyl-PC or -PE) in brain and myelin membranes and macrocyclic lipids with many ether-linked isoprenoid chains in archaebacteria and deep-sea microorganisms are typical examples of ether-linked phospholipids and their bilayer properties have been examined [58–61]. Next, we demonstrate the phase behavior of bilayers of dialkyl-PCs, which are PCs with two alkyl chains linking to the glycerol backbone by methylene groups (–CH_2_–) instead of carbonyl groups (>C=O). The substitution of the linkage influences the bilayer phase behavior remarkably. For example, the bilayer of dihexadecyl-PC (DHPC) undergoes the pre- and main transitions with increasing temperature under atmospheric pressure like the DPPC bilayer. Although the main transition is the transition from the P_β_′ phase to the L_α_ phase irrespective of the linkage difference, the pretransition of the DHPC bilayer is the transition from the L_β_I phase to the P_β_′ phase [62–64], and not the transition from the L_β_′ phase as observed in the DPPC bilayer. The DHPC bilayer induces the interdigitation by only hydration under atmospheric pressure because it weakens the interaction between adjacent lipid molecules. Since the pre- and main-transition temperatures of the DHPC and DPPC bilayers under atmospheric pressure are very close to each other, and both bilayers give similar thermodynamic quantities of the transition, it is difficult to distinguish the phase states by a method as DSC. In contrast to this, the phase states are definitely distinguished by pressurization of the both bilayers.

**Figure 8:**
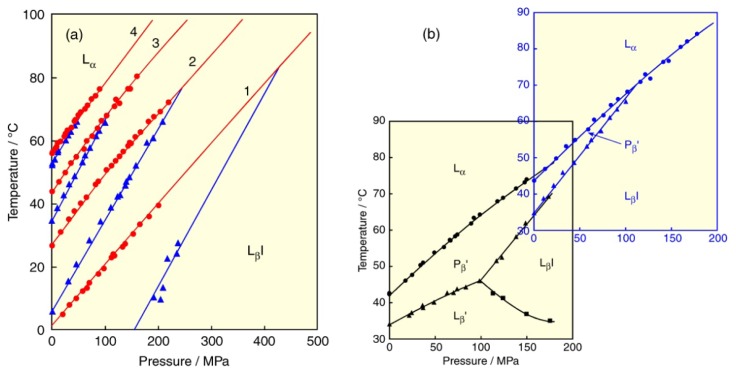
(**a**) Temperature–pressure phase diagrams of dialkyl-PC (ether-linked PC) bilayers: (1) *O*-C12PC, (2) *O*-C14PC, (3) *O*-C16PC (DHPC), (4) *O*-C18PC. Phase transitions: (blue) pre- (L_β_I/P_β_′) transition, (red) main transition. (**b**) Comparison of temperature–pressure phase diagram of DHPC bilayer (upper right diagram) with that of DPPC bilayer (lower left dagram).

Figure 8a illustrates the *T*–*p* phase diagrams constructed for the bilayers of four kinds of dialkyl-PCs, didodecyl-PC (*O*-C12PC), ditetradecyl-PC (*O*-C14PC), dihexadecyl-PC (*O*-C16PC (DHPC)) and dioctadecyl-PC (*O*-C18PC), respectively [65,66]. The difference in the pretransition between dialkyl- and diacyl-PCs appears in the slope of the pretransition curve on the phase diagrams: the slopes of the transition (L_β_′/P_β_′ transition) for the diacyl-PC bilayers are smaller than those of the main-transition curve, while those of the transition (L_β_I/P_β_′ transition) for the dialkyl-PC bilayers are conversely larger than them. Therefore, the pretransition and main transition curves intersect with each other at a certain pressure, then the P_β_′ phase disappears at high pressures above the intersection point and only the L_β_I/L_α_ transition occurs in the high-pressure region. This means that pressure stabilizes the L_β_I phase but destabilizes the P_β_′ and L_α_ phases. The effect of the alkyl chain length on the phase behavior synergizes the pressure effect, that is, the effect further extends the region of the L_β_I phase, while further shrinking the region of the P_β_′ and L_α_ phases. In addition, there is no pressure-induced phase in all the dialkyl-PC bilayers investigated.

The *T*–*p* phase diagrams of the dialkyl-PC bilayers are quite different from those of the diacyl-PC bilayers given in Figure 2 and Figure 3. The phase diagrams of the ether-linked PC bilayers are much simpler than the corresponding ester-linked PC bilayers [21] because of the lack of a pressure-induced phase. The substitution of an ether linkage for an ester linkage brings about the appearance of the L_β_I phase under ambient pressure. Overlapping the phase diagram of the DHPC bilayer in Figure 8a with that of the DPPC bilayer in Figure 2, we immediately notice that the phase behavior of the DPPC bilayer in a high-temperature and pressure region corresponds to the DHPC bilayers in the normal temperature and pressure region (Figure 8b). Furthermore, the overlap of the phase diagrams between both PC bilayers extends to the lower pressure region with an increase in hydrophobic chain length (data not shown, see ref. [66]), namely the elongation of hydrophobic chain length brings the phase behavior of both PC bilayers into closer relationship. When considering that membrane lipids of archaebacteria and deep-sea microorganisms are almost all ether-linked phospholipids and that an ether-linkage is chemically more stable than an ester linkage, it is so interesting that the phase behavior of the ether-linked PC bilayers bears a strong resemblance to that of the ester-linked PC bilayers under such extreme conditions as high temperature and pressure. The phase behavior of the dialkyl-PC bilayers means that the bilayers become more rigid than the diacyl-PC bilayer, and this behavior seems to be inconsistent with the fact at a glance that organisms living in an extreme condition have softer membranes. We speculate that the linkage-type change from the chemically stable ether-linkage at a high temperature and pressure to the chemically degradable ester-linkage is due to the environmental adaptation in the organic evolutionary process. However, a recent study on biological membranes of deep-sea microorganisms under high pressure [67] has shown that the membranes are not necessarily softer than those under atmospheric pressure, and do have, in fact, a rather rigid structure.

## 5. Effect of Solvent on the Phase Behavior of DPPC Bilayer Membrane

**Figure 9:**
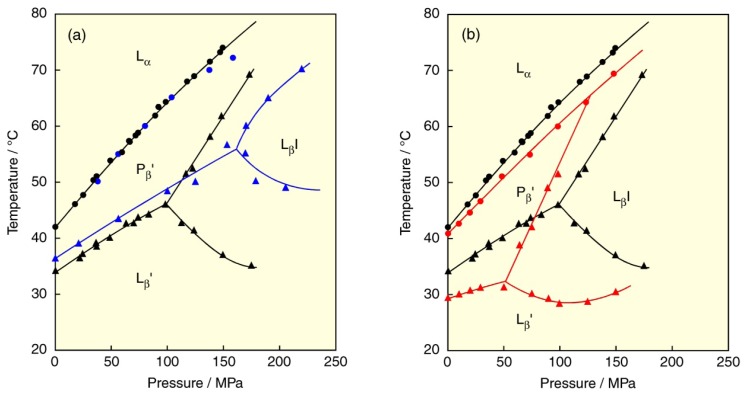
(**a**) Temperature–pressure phase diagrams of DPPC bilayer: (blue) in D_2_O, (black) in H_2_O. (**b**) Temperature–pressure phase diagrams of DPPC bilayer: (red) in 0.4 M ethanol solution, (black) in H_2_O. Phase transitions are the same as those in Figure 2b.

Finally, we describe the effect of a solvent on the PC bilayer. In Section 2, we mentioned the lyotropic phase transitions of the DPPC bilayer induced by hydration. Here we show how the difference in hydration force of a solvent affects the phase behavior of the DPPC bilayer. For this purpose, we used two solvents, one is heavy water (D_2_O) with stronger hydration force than light water (H_2_O), the other is an ethanol (EtOH) solution with weaker hydration force than H_2_O. Several kinds of studies with respect to solvent effects on the DPPC bilayer have been reported [68–71]. The constructed *T*–*p* phase diagrams of the DPPC bilayer in D_2_O and in 0.4 M ethanol solution are shown in Figure 9 in comparison with the diagram in H_2_O, respectively [72,73]. The main (P_β_′/L_α_) transition in D_2_O is almost consistent with that in H_2_O while it moves to the lower-temperature region to some extent at constant pressure in an EtOH solution than in H_2_O. In contrast to the behavior of the main transition, the transitions between the gel phases (pre- (L_β_′/P_β_′), L_β_′/ L_β_I and L_β_I/P_β_′) are markedly influenced by the substitution of solvent. This suggests that the substitution of solvent is more effective for the region of bilayer/water interface than for the hydrophobic core part of the bilayer.

By substituting D_2_O for H_2_O, the L_β_′/P_β_′ and L_β_′/L_β_I transition temperatures elevate, while the L_β_I/P_β_′ transition temperature depresses. These temperature changes can be explained as follows. The hydrogen bonds are stronger in D_2_O than in H_2_O [74,75], and this strong solvent interaction reduces an optimal area of the DPPC molecule in the bilayer. Because the area of the DPPC molecule in the gel phases increases in the order of the L_β_′ (0.39 nm_2_), P_β_′ (0.41 nm_2_) and L_β_I (0.78 nm_2_) phases [63,76], the stabilization of the gel phases in D_2_O in comparison with those in H_2_O is enhanced in the reverse order: L_β_′ > P_β_′ > L_β_I. The difference in stabilization among the gel phases results in the temperature elevation of the transition from left to right in the above inequality of the gel-phase stabilization, while it results in the temperature depression of the transition from right to left in the inequality. The change in stability between the gel phases by D_2_O produces the destabilization of the L_β_I phase and the region of the L_β_I phase moves to the high temperature and pressure region. Hence D_2_O suppresses the formation of the L_β_I phase. The bilayer interdigitation of phospholipid bilayers under high pressure was first observed for the DPPC bilayer in D_2_O by a method of small angle neutron scattering [12]; however, the effect of D_2_O on the phase behavior was not at all mentioned. We were later able to clarify the effect [72].

By contrast, the substitution of an EtOH solution for H_2_O causes a contrary situation: the L_β_′/P_β_′-and L_β_′/L_β_I-transition temperatures are depressed, while the L_β_I/P_β_′-transition temperature elevates. Since EtOH decreases the hydrogen bonding in the solution, the optimal area of the DPPC molecule in the bilayer increases. Then the stabilization of the gel phases in an EtOH solution in comparison with those in H_2_O is enhanced in the same order as the area in the gel phase: L_β_′ < P_β_′ < L_β_I, which brings about the opposite effect on the transition temperatures between the gel phases compared to D_2_O. Consequently, an EtOH solution promotes the formation of the L_β_I phase [68,73], that is, the L_β_I phase is stabilized and the region of the L_β_I phase moves to a low temperature and pressure region. The depression in the main-transition temperature in an EtOH solution compared with in D_2_O or in H_2_O suggests that a slight amount of EtOH molecules are incorporated into the hydrophobic core part of the bilayer and suppresses the chain melting.

## 6. Conclusions

In this review, we first described the phase behavior of the DPPC bilayer. We subsequently explained the various kinds of PCs with different molecular structures from DPPC by using the *T*–*p* phase diagrams, and finally illustrated the solvent effect on the phase behavior. To summarize the phase behavior of the PC bilayers, several phase states that the PC bilayers show are common to all PC bilayers, that is, the L_c_, L_β_ and L_α_ phases. It is characteristic of PCs with saturated acyl chains to exhibit the polymorphism of the gel phases such as L_β_′, P_β_′ and L_β_I phases, due to the steric hindrance between adjacent choline head groups of the PC molecules in the bilayer. The polymorphism is markedly affected by the chain length, chain asymmetry, linkage type and solvent substitution. It is also interesting that ester-linked PCs (diacyl-PCs) with an acyl-chain length of a carbon number between 14 and 21 induce the L_β_I phase under high pressure, while ether-linked PCs (dialkyl-PCs) induce the L_β_I phase under atmospheric pressure by only hydration. On the other hand, the introduction of double bonds into acyl chains produces the great stabilization of the L_α_ phase, and the effect is also markedly affected by the kind (*cis* or *trans*) and number of a double bond and the position of the unsaturated chain (*sn*-1 or *sn*-2). Consequently, we can say that the PC bilayers undergo various kinds of phase transitions, depending on hydration, temperature, pressure and solvent, and even a slight difference in the molecule structure of the PC molecule will yield a significant influence on the bilayer phase behavior.
